# A Conserved GA Element in TATA-Less RNA Polymerase II Promoters

**DOI:** 10.1371/journal.pone.0027595

**Published:** 2011-11-16

**Authors:** Martin Seizl, Holger Hartmann, Friederike Hoeg, Fabian Kurth, Dietmar E. Martin, Johannes Söding, Patrick Cramer

**Affiliations:** Department of Biochemistry and Gene Center, Center for Integrated Protein Science Munich (CIPSM), Ludwig-Maximilians-Universität (LMU) München, Munich, Germany; National Cancer Institute, United States of America

## Abstract

Initiation of RNA polymerase (Pol) II transcription requires assembly of the pre-initiation complex (PIC) at the promoter. In the classical view, PIC assembly starts with binding of the TATA box-binding protein (TBP) to the TATA box. However, a TATA box occurs in only 15% of promoters in the yeast *Saccharomyces cerevisiae*, posing the question how most yeast promoters nucleate PIC assembly. Here we show that one third of all yeast promoters contain a novel conserved DNA element, the GA element (GAE), that generally does not co-occur with the TATA box. The distance of the GAE to the transcription start site (TSS) resembles the distance of the TATA box to the TSS. The TATA-less *TMT1* core promoter contains a GAE, recruits TBP, and supports formation of a TBP-TFIIB-DNA-complex. Mutation of the promoter region surrounding the GAE abolishes transcription *in vivo* and *in vitro*. A 32-nucleotide promoter region containing the GAE can functionally substitute for the TATA box in a TATA-containing promoter. This identifies the GAE as a conserved promoter element in TATA-less promoters.

## Introduction

In eukaryotes, transcription of protein-coding genes relies on RNA polymerase (Pol) II, the general transcription factors (GTFs) TFIIB, -D, -E, -F, and –H, and coactivators such as Mediator and the Spt-Ada-Gcn5 acetyltransferase complex (SAGA) [1], [2]. During activation, gene-specific transcription factors recruit coactivator complexes and TFIID, thereby facilitating pre-initiation complex (PIC) formation at the promoter. A subunit of TFIID, the TATA box-binding protein (TBP), binds the TATA box, which is located upstream of the transcription start site (TSS), and nucleates PIC assembly [3], [4]. However, TATA boxes with the consensus sequence TATAWAWR occur in only 13% of yeast promoters [5], and 10% of human promoters [6].

Since TATA-less promoters require TBP for function [7], [8], [9], and bind TFIID [10], alternative pathways for PIC assembly were proposed [1], [11]. In metazoans, many TATA-less promoters contain a downstream promoter element (DPE) and an initiator (INR), which interact with components of TFIID to facilitate PIC assembly [12], [13]. Although the GTFs are highly conserved throughout eukaryotes, alternative core promoter elements could not be identified in yeast. A TATA box is not strictly required for TFIID-dependent activity of a yeast model promoter [14]. Indeed, recent studies of the TATA-less yeast RPS5 promoter showed that functionally redundant AT-rich stretches within the core promoter region promote TFIID-dependent transcription [15].

Based on these observations, we hypothesized that TATA-less core promoters contain DNA elements that are functionally similar to the TATA box in promoting PIC assembly and Pol II transcription. Here we used a combination of bioinformatics, *in vivo* reporter gene assays, and *in vitro* biochemistry to identify and functionally characterize a region in TATA-less yeast core promoters that is bound by TBP, required for Pol II transcription and contains a novel conserved promoter element, the GA element, or GAE.

## Results

### Many TATA-less yeast promoters contain a conserved GAE

In contrast to higher eukaryotes, where the distance between the TATA box and the TSS is fixed at around 30 nucleotides, a variable distance of 40−120 nucleotides is observed in yeast, apparently due to a TSS scanning mechanism [16]. This has hampered bioinformatic discovery of core promoter elements other than the TATA box in yeast. To systematically search for a core promoter motif that could be functionally similar to the TATA box in TATA-less yeast promoters, we defined four criteria: (1) The motif should peak within a core promoter window of −110 to −50 nucleotides relative to the TSS. (2) The motif occurrence should be anti-correlated to the TATA box. (3) The motif should be frequent in this region in TATA-less promoters. (4) The motif should be highly conserved.

**Figure 1:**
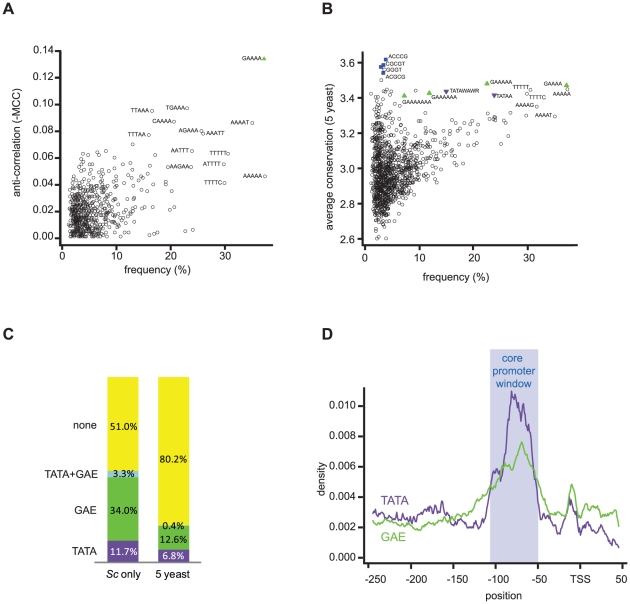
Many TATA-less yeast promoters contain a conserved GAE. (A) Anti-correlation between different 5-mers and the TATA box in *Sc* core promoters. GAE is marked by a green triangle. (B) Frequency of DNA 5-mers and their conservation between five closely related yeast species (*Sc, S. paradoxus*, *S. mikatae*, *S. kudriavzevii*, *S. bayanus)* in the core promoter window (−110 to −50 relative to TSS) of 3711 Pol II promoters. For comparison, TATA consensus (TATAWAWR) and GAE-related sequences are shown. TATA-related sequences are marked by a purple triangle, GAE-related sequences are marked by a green triangle, transcription factor binding sites are marked by a blue square, and other 5-mers are marked by an open circle. (C) Percentage of *Sc* core promoters containing the TATA box, GAE, both elements or none of the elements with and without conservation between five closely related yeast species (left and right column, respectively). (D) Occurrence of the TATA box and GAE in 3711 *Sc* Pol II promoters aligned to the TSS. Core promoter window used for bioinformatics analysis is highlighted by a blue box.

For each of the 1024 possible 5-mers, we calculated the frequency of occurrence within the core promoter window and the Matthews correlation coefficient with the TATA box (consensus TATAWAWR). This search identified a DNA element comprising one guanine followed by four adenines (GAAAA) as being most highly anti-correlated to the TATA box (Figure 1A, criterion 1 and 2). We refer to this novel element as GA element (GAE). It displays the second highest frequency of occurrence in the core promoter window (Table S1), only trumped in frequency by a subsequence of its longer version GAAAAA (criterion 3). We next analyzed the conservation of all 5-mers in the core promoter window among five closely related yeast species (*Sc, S. paradoxus*, *S. mikatae*, *S. kudriavzevii*, *S. bayanus).* We allowed for a shift of up to +/− 3 nucleotides between motifs in the multiple sequence alignments. Again, the GAE stands out as being the most highly conserved of all the more frequent 5-mers (Figure 1B, criterion 4). Its degree of conservation is slightly higher than that of the full and partial TATA box consensus (TATAWAWR and TATAA) and only a little lower than the more rare consensus binding sites of transcription factors Reb1 (ACCCG) and Mbp1 (ACGCG). Therefore, the GAE meets all four criteria defined above.

*Sc* promoters containing only a GAE are three times more abundant than promoters containing only a TATA box (34% vs. 11.7%, respectively, Figure 1C). When filtered by conservation between the five closely related yeast species, 12.6% of promoters still contained the GAE, whereas 6.8% contained a TATA box (Figure 1C). The GAE is 3.4 times more frequent than expected from a mononucleotide background model calculated on the same region. This exceeds the TATA box and an A-stretch of length five, which are 3.3 times and 2.0 times more frequent, respectively. The presence of a GAE is anti-correlated with the presence of a TATA box (Figure 1C). Only 0.4% of all promoters contain both elements (Figure 1C). The GAE can be present in more than one copy in the core promoter region (Figure S1) and can contain additional adenines at the 3’end (Figure S2). When promoters are aligned to the TSS, the GAE peaks in the same region as the TATA box (Figure 1D). The similar position of the TATA box and the GAE with respect to the TSS, and the mutual exclusion of these two elements from core promoters suggested a functional similarity of both elements during PIC assembly.

### A TATA-less yeast promoter that functions in vitro

**Figure 2:**
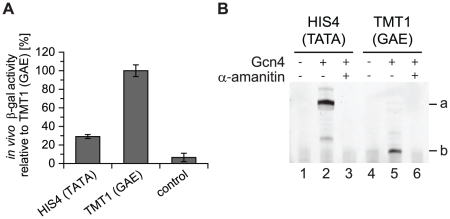
A TATA-less yeast promoter that functions in vitro. (A) Promoter activity in an *in vivo* β-galactosidase reporter gene assay of *HIS4* and *TMT1 Sc* promoters. Negative control is background signal of the reporter plasmid without a promoter. (B) Promoter activity in an *in vitro* transcription assay of *Sc HIS4* and *Sc TMT1* promoter using yeast nuclear extracts. *In vitro* transcription was carried out with and without recombinant Gcn4 activator. To test Pol II specificity of transcription, 0.04 mM α-amanitin was added. Specific transcripts from the *HIS4* TSS and *TMT1* TSS are marked by a and b, respectively.

To functionally characterize the GAE *in vivo* and *in vitro*, we chose the *Sc TMT1* promoter, a TATA-less promoter with a single GAE in the core promoter region. For comparison, we used the *Sc HIS4* promoter, which contains a TATA box and is widely used for *in vitro* transcription assays [4], [17], [18], [19]. Both *TMT1* and *HIS4* are involved in amino acid biosynthesis and are regulated by the activator Gcn4. We tested both promoters in a β-galactosidase reporter gene assay, which measures promoter strength *in vivo*. Both promoters were active under standard growth conditions (Figure 2A). We next examined whether the promoters also supported Gcn4-activated transcription *in vitro*. Indeed, not only the TATA-containing *HIS4* promoter but also the TATA-less *TMT1* promoter was active (Figure 2B, lanes 2 and 5). Promoter activity depended on Pol II, since the Pol II-specific inhibitor α-amanitin abolished transcription (Figure 2B, lanes 3 and 6). These results establish the *TMT1* promoter as the first native TATA-less promoter in yeast that is active in an activator-dependent *in vitro* transcription assay.

### The GAE-containing region is required for promoter function

**Figure 3:**
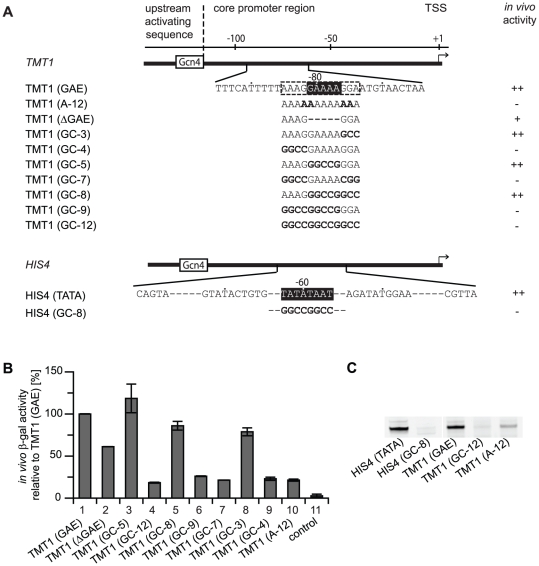
GAE-containing region is required for promoter function. (A) Schematic depiction of the TATA-less *TMT1* and the TATA-containing *HIS4* promoter. TATA box and bioinformatically defined GAE are marked by a black box. The 12-nucleotide GAE-containing region is marked by a dashed line. Promoter mutants are shown below the sequences. Activity of each variant in *in vivo* β-galactosidase reporter gene assays is shown (wildtype activity (++), decreased activity (+) and severely decreased activity (−)). (B) Determination of the core promoter region required for *TMT1* promoter function. Activities of wild type *TMT1* and several mutants in the GAE-containing region in an *in vivo* β-galactosidase reporter gene assay are shown. Negative control is background signal of the reporter plasmid without a promoter. (C) Promoter activities in an *in vitro* transcription assay using a nucleosome-free DNA template and yeast nuclear extracts.

To test whether the GAE is required for *TMT1* promoter function, we generated several promoter mutants (Figure 3A) and tested their activities in β-galactosidase reporter gene assays. Deletion of the bioinformatically defined 5-nucleotide GAE had a moderate negative effect on promoter activity (Figure 3B, lane 2). Whereas replacing the 5-nucleotide GAE with GGCCG had no apparent defect (Figure 3B, lane 3), mutating the 12-nucleotide GAE-containing region (four and three nucleotides up- and downstream, respectively) strongly impaired promoter activity (Figure 3B, lane 4). Screening of additional promoter mutants revealed a strong dependence of promoter function on the four nucleotides directly upstream of the GAE (Figure 3B, lane 9).

Activity of the *TMT1* promoter was also strongly decreased when the guanines in the 12-nucleotide GAE-containing region were changed to adenines (Figure 3B, lane 10), generating a poly(dA) stretch. A similar defect in promoter activity was observed in activated transcription assays using a nucleosome-free DNA template and yeast nuclear extracts (Figure 3C) *in vitro*. Thus the GAE-containing region is functionally distinct from previously described poly(dA) stretches, which are proposed to occlude nucleosomes from promoters [20], [21]. These results suggest that the role of the GAE-containing region is not related to nucleosome-dependent promoter accessibility. The defects of the GAE deletion, the mutations in the flanking regions, and the polyA mutation suggest that *TMT1* promoter function is strongly dependent on the sequence context in the GAE-containing region.

### A TATA-less promoter is bound by TBP

**Figure 4:**
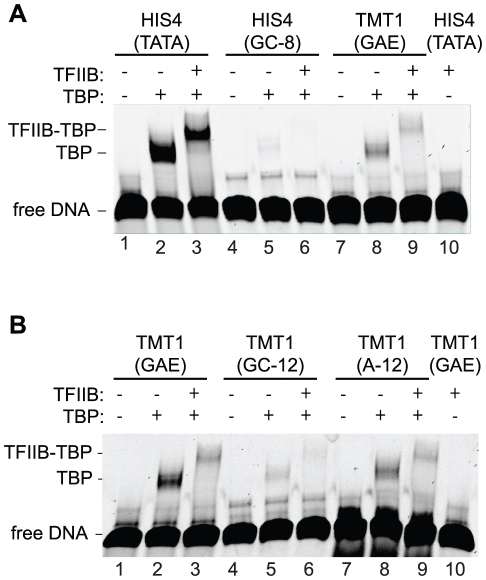
The TATA-less TMT1 core promoter is bound by TBP. (A) and (B) Electrophoretic mobility shift assay. A 5’-Cy5-labeled 40 bp double stranded DNA of the respective promoter construct was incubated with recombinant TBP_core_ alone or TBP_ core_ and TFIIB. The protein-DNA complex was separated from free DNA by native polyacrylamide gel electrophoresis. Free DNA and bound protein-DNA complexes are indicated.

Because the GAE and the TATA box occur at similar distances from the TSS, we tested whether the GAE-containing promoter region binds TBP. We used an electrophoretic mobility shift assay (EMSA) and fluorescently labeled DNA encompassing 40 base pairs of *HIS4* and *TMT1* promoter DNA. TBP bound *HIS4* DNA as expected, but also bound the TATA-less *TMT1* DNA (Figure 4A, lane 2 and 8). Both promoter DNAs could also form a stable complex with TBP and TFIIB (Figure 4A, lane 3 and 9). The observed binding was specific to double stranded DNA (Figure S3). As expected, TBP and TFIIB binding was strongly decreased by mutating the TATA box of *HIS4* (Figure 4A, lane 5 and 6). Mutating the 12-nucleotide GAE-containing region also impaired TBP and TFIIB binding (Figure 4B, lane 5 and 6). TBP and TFIIB binding was not affected when guanines in the 12-nucleotide GAE-containing region were replaced with adenines (Figure 4B, lane 8 and 9). These results show that a stretch of adenines is sufficient for TBP binding, but not for promoter activity. The guanines in and around the GAE thus appear to have a function distinct from TBP binding. This is consistent with the observation that TBP binding does not necessarily correlate with promoter activity [22], [23].

### The GAE-containing region functionally substitutes for the TATA box

**Figure 5:**
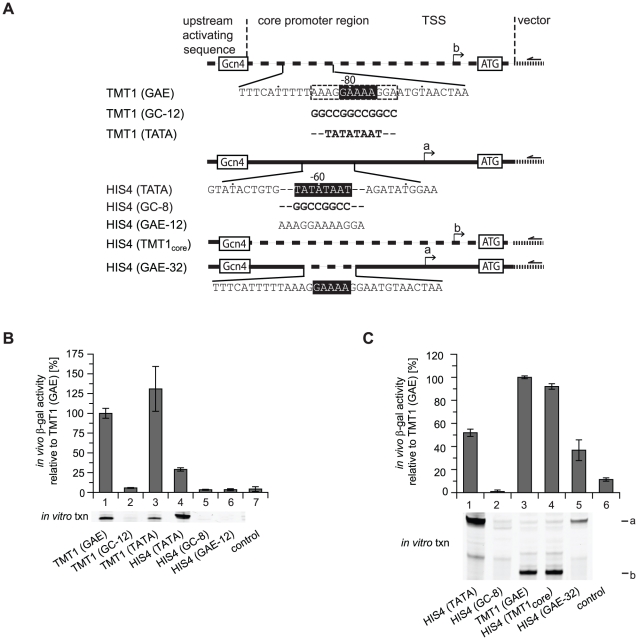
The GAE-containing region functionally substitutes for the TATA box. (A) Schematic depiction of promoter substitution constructs of the TATA-less *TMT1* and the TATA-containing *HIS4* promoter. TATA box and bioinformatically defined GAE are marked by a black box. Solid and dashed lines represent *HIS4* and *TMT1* promoter sequences, respectively. TSS of *HIS4* and *TMT1* are labeled with a and b, respectively. Half-arrows mark primer annealing sites for primer extension reaction. (B) Promoter activity in an *in vivo* β-galactosidase reporter gene assay and *in vitro* transcription (txn) assay of the wild type (lane 1 and 4) and mutated *TMT1* and *HIS4* promoter. Mutations as indicated in Figure 5A. Negative control (lane 7) is background signal of the reporter plasmid without a promoter. (C) Promoter activity in an *in vivo* β-galactosidase reporter gene assay and *in vitro* transcription (txn) assay of the wild type (lane 1 and 3) and promoter substitution constructs as indicated in Figure 5A. Negative control (lane 6) is background signal of the reporter plasmid without a promoter.

To further investigate the potential functional similarity of the GAE-containing region and the TATA box, we performed promoter substitution experiments *in vivo* and *in vitro*. We found that the *HIS4* TATA box can functionally replace the 12-nucleotide GAE-containing region in the *TMT1* promoter (Figure 5A, lane 3). This substitution does not impair binding of TBP and TFIIB (Figure S4A, lanes 5 and 6). In contrast, the 12-nucleotide GAE-containing region is neither sufficient for functional replacement of the TATA box in the *HIS4* promoter (Figure 5A, lane 6) nor for binding of TBP and TFIIB (Figure S4B, lane 5 and 6). Similar observations were made when replacing the TATA box in the *SER3* yeast promoter by the 12-nucleotide GAE-containing region (Figure S4C).

To determine a minimal region in the *TMT1* promoter that could substitute part of the *HIS4* promoter without loss of activity, we tested several *HIS4-TMT1* fusion constructs (Figure 5B). We found that the *TMT1* core promoter fused to the *HIS4* upstream activating sequence is active both *in vivo* and *in vitro* (Figure 5C, lane 4). This suggests that regions surrounding the 12-nucleotide GAE-containing region are required for function. Indeed, inserting a 32-nucleotide GAE-containing region (14 and 13 nucleotides up- and downstream of the GAE, respectively) could maintain partial *HIS4* activity (Figure 5C, lane 5). Including even longer up- or downstream *TMT1* sequences did not increase activity (Figure S5). This is consistent with our observation that sequence context is critical for *TMT1* activity (Figure 3B). This indicates that the AT-rich sequences flanking the GAE in the *TMT1* promoter are also important for activity.

## Discussion

Here we describe a novel core promoter element in yeast, the GA element, or GAE, which is found almost exclusively in TATA-less promoters. Similar to TATA-containing promoters, the GAE-containing *TMT1* promoter is bound by TBP and supports formation of a TBP-TFIIB-DNA complex. Together with the anti-correlation with canonical TATA boxes, this suggests a functional similarity of the TATA box and the GAE-containing region. The mutual exclusion of TATA box and GAE appears not to be the result of an inhibitory role of GAE. Insertion of the GAE up- or downstream of the *HIS4* TATA box had no effect on promoter function (Figure S6). We demonstrate that *TMT1* promoter function is highly dependent on sequence context. The GAE-flanking region and the guanine residues in the 12-nucleotide GAE-containing region are crucial for promoter function. Similarly, previous studies have shown that also TATA box function and TBP-TATA binding is influenced by TATA-flanking sequences [24]. In contrast to the TATA box, the GAE can be present in more than one copy in a promoter. Similarly, a study of the TATA-less *RPS5* promoter demonstrated the presence of multiple, functionally redundant AT-rich sequences, which were shown to recruit TFIID [15].

Recent genome-wide studies indicated that activation of TATA-less genes is dominated by TFIID [5], [25]. Consistently, a detailed study of the TATA-less *TUB2* promoter had demonstrated a strong TFIID dependence [26]. *TUB2* activity was severely reduced in temperature-sensitive TFIID mutant backgrounds. Insertion of a canonical TATA box at -55 relative to the TSS could alleviate this defect and restore promoter function. Intriguingly, when we inspected the *TUB2* sequence, we found a conserved GAE at the exact point of insertion. This is consistent with our finding that the GAE is mainly found in TFIID-dominated promoters (Figure S7).

Several studies of poly(dA:dT) tracts in individual model promoters indicated a role in promoter function that is linked to nucleosome positioning [20], [21], [27], [28], [29]. Further, a recent theoretical genome-wide study described G/C-capped poly(dA:dT) tracts associated with the nucleosome-free region of TATA-less promoters in yeast [30]. These tracts were suggested to define the center of the nucleosome-free region. However, a distinction of a direct effect on nucleosome positioning and indirect effects, such as recruiting chromatin remodelers or the transcription machinery, could not be made. Our *in vitro* transcription experiments done on a nucleosome-free DNA template demonstrate a direct role of the GAE-containing region in transcription, and suggest a function independent of nucleosome positioning. Consistent with our findings, the deletion of a poly(dA:dT) tract in the *ILV1* promoter decreased transcription significantly without affecting nucleosome organization *in vivo* [28]. Since the GAE described here appears to overlap with a part of the previously described poly(dA:dT) tracts, those tracts that fall in the described core promoter window may be redefined as GAE.

## Materials and Methods

### Bioinformatics

The TSS used for our analysis were generated by merging the cDNA data set of Miura et. al [31] and the 5’-SAGE data of Zhang and Dietrich [32]. In both sets the TSS was considered to be the position with the highest tag count in a window of at most 300 bp upstream of a gene. If a gene has tag counts in both data sets, the TSS of the Miura set was used. The resulting data set consists of 3711 TSSs for annotated ORFs. For co-occurrence analysis, the Matthews Correlation Coefficient (MCC) was calculated for all 5-mers with respect to the TATA box consensus site (TATAWAWR) using the following formula.
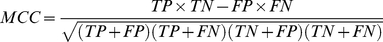
where TP stands for regions with both the 5-mer and the TATA box present, TN stands for regions with no motif present, FP stands for regions with only the TATA box present, and FN stands for regions with only the 5-mer present. Only occurrences were used that started in the TATA box region, i.e. −110 to −50 relative to the TSS. The conservation score was then calculated as the mean number of conserved nucleotides per motif position. This score ranges between 0 (all positions are mutated) and 4 (every motif position is conserved in all four related species), since five related yeast species (*Sc*, *S. paradoxus*, *S. mikatae*, *S. kudriavzevii*, *S. bayanus*) were used. For calculating the number of mutations, the start position in a window of +/−3 bp with the least number of mutations to the motif in *Sc* in every species was used.

### Reporter gene assay

The native promoter sequences of *HIS4* (428 bp upstream to 24 bp downstream of the start codon) and *TMT1* (273 bp upstream to 24 bp downstream of the start codon) and their mutant variants were cloned between *Hind*III/*Bam*HI into a pRS315 plasmid with the lacZ gene inserted between *Not*I/*Sal*I. Reporter plasmids were transformed into BY4741 wild type yeast cells and grown in biological duplicates in SD (–leu) medium to an OD_600_ of 0.5–1.0. β-galactosidase levels were determined using the Yeast β-galactosidase Assay Kit (Thermo Scientific, #75768).

### In vitro transcription

Nuclear extracts were prepared from 3 l of BY4741 wild type yeast culture as described (www.fhcrc.org/science/basic/labs/hahn) 33]. *In vitro* transcription and analysis by primer extension were performed as described 33]. Primer extension for all constructs was done using the same 5’-Cy5-labeled oligonucleotide (5’-TTCACCAGTGAGACGGGCAAC). For activated transcription 200 ng of recombinant full-length Gcn4 was added. Template plasmids were generated by inserting the respective promoter sequence as described above in pBluescript KS+ with *Hind*III and *Bam*HI. The *Sc* Gcn4 gene was inserted into vector pET21a with *Nco*I and *Sac*I introducing an N-terminal hexahistidine tag. Transformed *E. coli* BL21 (DE3) RIL cells (Stratagene) were grown in LB medium at 37°C to an OD_600_ of 0.5. Expression was induced with 0.5 mM IPTG for 16 h at 18°C. For protein purification, cells were lysed by sonication in buffer A (20 mM Tris/HCl pH 8.0 25°C, 500 mM NaCl, 5 mM DTT). After centrifugation, the supernatant was loaded twice onto a 2 ml Ni-NTA column (Qiagen) equilibrated with buffer A. The column was washed three times with 10 column volumes (CV) of buffer A containing 20 mM imidazole. Bound protein was eluted with 10 CV of buffer A containing 500 mM imidazole. Protein was further purified by cation exchange chromatography using a HiTrap SP column (GE Healthcare) equilibrated with buffer B (20 mM HEPES pH 7.5 25°C, 10% Glycerol, 1 mM EDTA, 2 mM DTT). The protein was eluted with a linear gradient of 10 CVs from 0 to 1 M NaCl in buffer B. After concentration, the sample was applied to a Superdex 200 size exclusion column (Amersham) equilibrated with buffer C (20 mM HEPES pH 7.5 25°C, 150 mM KAcetate, 10% Glycerol, 1 mM EDTA, 2 mM DTT). The sample was concentrated to approximately 0.5 mg/ml, flash frozen in small aliquots in liquid nitrogen, and stored at −80°C. All buffers contained a protease inhibitor cocktail.

### Electrophoretic mobility shift assay

Electrophoretic mobility shift assays (EMSA) were performed as described 34], with minor modifications. Templates were generated by annealing the complimentary PAGE-purified oligonucleotides (coding strand was 5’-Cy5-labeled). The binding reaction contained 3 nM of 40 bp 5’-Cy-5-labeled dsDNA, 300 nM TBP_core_ and 3 µM TFIIB, 1 mM DTT, 50 µg/ml Heparin in 1x binding buffer (4% glycerol, 4 mM Tris-HCl pH 8.0 23°C, 60 mM KCl, 5 mM MgCl_2_, 100 µg/ml BSA, 0.1% Tween 20) in a total reaction volume of 20 µl. Recombinant TBP_core_ and TFIIB were expressed in *E. coli* and purified as described 35]. Proteins were diluted in dilution buffer (20 mM Tris pH 7.9 23°C, 150 mM KCl, 1 mM DTT, 10% Glycerol, 50 µg/ml BSA). Samples were incubated for 30 min at 18°C and loaded on a 6% native polyacrylamide gel (acrylamide:bisacrylamide 60∶1, 190 mM glycine, 25 mM Tris pH 8.3 23°C with acetic acid, 0.5 mM DTT, 0.1% APS, 1% TEMED). The gel was pre-run for five min and then run at 160V for 15 min at room temperature in 190 mM glycine, 25 mM Tris pH 8.3 23°C. Gels were analyzed with a typhoon scanner FLA9400 and ImageQuant Software (GE Healthcare). The oligos used for the assay had the following sequence (only coding strand is shown):

HIS4 (TATA), 5’-ACAGTAGTATACTGTGTATATAATAGATATGGAACGTTAT;

HIS4 (GC-8), 5’-ACAGTAGTATACTGTGGGCCGGCCAGATATGGAACGTTAT;

HIS4 (GAE), 5’-TAGTATACTGTGAAAGGAAAAGGAAGATATGGAACGTTAT;

TMT1 (GAE), 5’-ATTTTCATTTTTAAAGGAAAAGGAATGTAACTAATTTAGT;

TMT1 (GC-12), 5’-ATTTTCATTTTTGGCCGGCCGGCCATGTAACTAATTTAGT;

TMT1 (TATA), 5’-ATTTTCATTTTTTATATAATATGTAACTAATTTAGT;

TMT1 (A-12), 5’- ATTTTCATTTTTAAAAAAAAAAAAATGTAACTAATTTAGT

## Supporting Information

Figure S1 **The GAE can occur in multiple copies.** Fraction of sequences with the TATA box (purple) and GAE (green) in at least a given number of multiple occurrences per sequences in 3711 *Sc* promoters aligned at the TSS.
(EPS)

Figure S2 **Frequency of the GAE with different length of A-tracts in 3711** ***Sc*** **core promoters.**
(EPS)

Figure S3 **Only double stranded templates are bound by TBP and TFIIB.** Electrophoretic mobility shift assay. A 5’-Cy5-labeled 40 bp single (ssDNA) and double stranded (dsDNA) templates were incubated with recombinant TBP_core_ alone or TBP_ core_ and TFIIB. The protein-DNA complex was separated from free DNA by native polyacrylamide gel electrophoresis. Free DNA and bound protein-DNA complexes are indicated.
(EPS)

Figure S4 **GAE function depends on promoter context.** (A) and (B) Electrophoretic mobility shift assay. A 5’-Cy5-labeled 40 bp double stranded DNA of the respective promoter construct was incubated with recombinant TBP_core_ alone or TBP_ core_ and TFIIB. The protein-DNA complex was separated from free DNA by native polyacrylamide gel electrophoresis. Free DNA and bound protein-DNA complexes are indicated. (C) Promoter activity in an *in vivo* β-galactosidase reporter gene assay and *in vitro* transcription (txn) assay of the wild type and mutated *Sc SER3* promoter. The 8-nucleotide TATA box of the *SER3* promoter was substituted by the 12-nucleotide GAE-containing region of the *TMT1* promoter.
(EPS)

Figure S5 **The GAE-containing region can functionally replace the TATA box.** (A) Schematic depiction of promoter substitution constructs of the TATA-less *TMT1* and the TATA-containing *HIS4* promoter. TATA box and bioinformatically defined GAE are marked by a black box. Solid and dashed lines represent *HIS4* and *TMT1* promoter sequences, respectively. TSS of *HIS4* and *TMT1* are labeled with a and b, respectively. Half-arrows mark primer annealing sites for primer extension reaction. (B) Promoter activity in an *in vivo* β-galactosidase reporter gene assay and *in vitro* transcription (txn) assay of the wild type and mutated *HIS4* and *TMT1* promoter. Negative control (lane 8) is background signal of the reporter plasmid without a promoter. Specific transcripts from the *HIS4* TSS and *TMT1* TSS are marked by a and b, respectively.
(EPS)

Figure S6 **GAE has no inhibitory effect on the TATA-containing HIS4 promoter.** Promoter activity in an *in vivo* β-galactosidase reporter gene assay of the wild type and mutated HIS4 promoter. Promoter substitution constructs as indicated on the right. Negative control is background signal of the reporter plasmid without a promoter.
(EPS)

Figure S7 **The GAE anti-correlates with SAGA-dominated genes.** Percentage of *Sc* core promoters containing TATA box, GAE, both elements or none of the elements in SAGA- and TFIID dominated *Sc* promoter classes [5], [25].
(EPS)

Table S1 **All** ***Sc*** **promoters containing a bioinformatically defined GAE.**
(XLS)
